# Nutritional Intake, Sports Nutrition Knowledge and Energy Availability in Female Australian Rules Football Players

**DOI:** 10.3390/nu11050971

**Published:** 2019-04-28

**Authors:** Dominique Condo, Rachel Lohman, Monica Kelly, Amelia Carr

**Affiliations:** 1 Centre for Sport Research, School of Exercise and Nutrition Sciences, Faculty of Health, Deakin University, 221 Burwood Highway, Burwood, Melbourne, VIC 3125, Australia; rlohma@deakin.edu.au (R.L.); amelia.carr@deakin.edu.au (A.C.); 2 Institute for Physical Activity and Nutrition, School of Exercise and Nutrition Sciences, Faculty of Health, Deakin University, 221 Burwood Highway, Burwood, Melbourne, VIC 3125, Australia; 3 Geelong Football Club, GMHBA Stadium, Geelong, VIC 3125, Australia; mkelly@geelongcats.com.au

**Keywords:** carbohydrate intake, team sports, female athletes, nutritional recommendations, nutrition knowledge

## Abstract

This study aimed to assess nutritional intake, sports nutrition knowledge and risk of Low Energy Availability (LEA) in female Australian rules football players. Victorian Football League Women’s competition (VFLW) players (n = 30) aged 18–35 (weight: 64.5 kg ± 8.0; height: 168.2 cm ± 7.6) were recruited from Victoria, Australia. Nutritional intake was quantified on training days using the Automated 24 h Dietary Assessment Tool (ASA24-Australia), and sports nutrition knowledge was measured by the 88-item Sports Nutrition Knowledge Questionnaire (SNKQ). The risk of LEA was assessed using the Low Energy Availability in Females Questionnaire (LEAF-Q). Daily mean carbohydrate intake in the current investigation was 3 g⋅kg^−1^⋅d^−1^, therefore, below the minimum carbohydrate recommendation for moderate exercise of approximately one hour per day (5–7 g⋅kg^−1^⋅d^−1^) and for moderate to intense exercise for 1–3 h per day (6–10 g⋅kg^−1^⋅d^−1^) for 96.3% and 100% of players, respectively. Daily mean protein intake was 1.5 g⋅kg^−1^⋅d^−1^, therefore, consistent with recommendations (1.2–2.0 g⋅kg^−1^⋅d^−1^) for 77.8% of players. Daily mean calcium intake was 924.8 mg⋅d^−1^, therefore, below recommendations (1000 mg⋅d^−1^) for 65.5% of players, while mean iron intake was 12.2 mg⋅d^−1^, also below recommendations (18 mg⋅d^−1^) for 100% of players. Players answered 54.5% of SNKQ questions correctly, with the lowest scores observed in the section on supplements. Risk of LEA was evident in 30% of players, with no differences in carbohydrate (*p* = 0.238), protein (*p* = 0.296), fat (*p* = 0.490) or energy (*p* = 0.971) intakes between players at risk of LEA and those not at risk. The results suggest that female Australian rules football players have an inadequate intake of carbohydrate and calcium and low sports nutrition knowledge. Further investigation to assess the risk of LEA using direct measures is required.

## 1. Introduction

In 2017, the Australian Football League Women’s (AFLW) competition commenced. In addition to the elite AFL competition, Australian rules football is comprised of numerous sub-elite competitions including the Victorian Football League (VFL) and regional leagues [1,2]. Prior to the initiation of the AFLW, there was no elite-level competition available to female Australian rules football players, and, therefore, many current players have transferred from other sports, including basketball and soccer [3]. Although AFLW in Australia is rapidly evolving, many AFLW and Victorian Football League Women’s (VFLW) players have experienced minimal elite sport experience within a structured training program [3]. It has been established that differences exist between the male elite AFL and sub-elite VFL competition, in terms of increased match intensity, greater running distance and overall game speeds for AFL players [2,4]. However, these differences may not exist between AFLW and VFLW players because the competitions are similar, both characterised by shorter quarters (up to 20 min), no limit on the number of rotations, and players’ part-time status [3].

The nutritional requirements of Australian rules football players vary throughout the season [5], which may impact on macronutrient consumption, including appropriate carbohydrate, protein and total energy intake. Current daily carbohydrate recommendations for moderate- to high-intensity exercise (1–4 h duration) range between 5 and 12 g⋅k^−1^ body mass (BM) [6]. In the context of Australian rules football, moderate- to high-intensity exercise between 1 and 3 h is performed during pre-season training, in-season main training sessions and during a match. Carbohydrate requirements are reduced during low-intensity exercise of up to one hour (3–5 g⋅k^−1^⋅d^−1^ BM) [6]. Contrary to recommendations, previous studies have reported relatively low carbohydrate intakes in male AFL players (<5 g⋅kg^−1^⋅d^−1^) [5,7,8], and similar intakes have been identified in Australian female athletes from a range of individual and team sports [9]. While the recommended carbohydrate intake for high-intensity exercise is well established, the consistently reported low carbohydrate intake in team sport athletes may suggest that the physical demands of these sports require less carbohydrate than current recommendations. In contrast, athletes tend to exceed the recommended protein intake [9]. Protein requirements for athletes are higher than those for the general population (0.8–1.0 g⋅kg^−1^⋅d^−1^ [10]) to assist with muscle protein synthesis and recovery, with the current recommendations specifying that athletes consume 1.2–2 g⋅kg^−1^⋅d^−1^, depending on training program goals and overall energy budgets [6]. The average reported protein intake for Australian female athletes is 1.6 g⋅kg^−1^⋅d^−1^ [9]. At present, recommendations for micronutrient intake in athletes are consistent with general population health guidelines [10]. However, it is likely that certain athletes may have an increased requirement for specific micronutrients, including vitamins B for athletes who restrict their energy intake, which is common in female athletes [9], iron for endurance athletes and calcium and vitamin D for amenorrheic females [6,9]. The current literature assessing micronutrient intake in female athletes is limited, with one Australian study reporting that mean micronutrient intake met the relevant Australian Nutrient Reference Values [10]. Despite this, a significant proportion of individual athletes in this study failed to meet the general population health recommendations, in particular for folate (44%), calcium (22%), iron (19%) and magnesium (15%) [9].

Nutrition education is one strategy used to assist athletes to consume an adequate diet [11]. Athletes with higher nutrition knowledge are more likely to consume more fruit, vegetables and carbohydrate-rich foods than those with low nutritional understanding [12], which suggests that sports nutrition knowledge may be associated with appropriate dietary intake. Women have been reported to have a higher level of nutrition knowledge than men, with a recent study reporting that Australian female athletes had a higher total knowledge score than other participants [13]. In male Australian rules footballers, three studies have reported poor sports nutrition knowledge, with only approximately half of the questions being answered correctly [7,8,14]. Currently, sports nutrition knowledge in female football players who have not been training within an elite program or had regular exposure to nutrition support is unknown.

Energy availability (EA) is defined as the ingested energy remaining for all other metabolic processes after the energy cost of training has been subtracted [15]. Low EA (LEA) occurs when the amount of energy available for basic physiological functions becomes insufficient, which, if continued, may lead to a significant health risk for female athletes [16]. LEA may be present with or without a clinical eating disorder, with the causes often being unintentional and due to increased energy expenditure without an increase in energy intake [17]. Directly measuring EA can be challenging as it requires the quantification of 24 h energy intake and expenditure over a period of time, which is often impractical and burdensome for athletes. Furthermore, there is currently no gold standard approach for the measurement of exercise energy expenditure, as many tools used in an applied context, including accelerometers, underestimate energy expenditure at high exercise intensities [18]. Because of the challenges associated with directly measuring EA, the validated Low Energy Availability in Females Questionnaire (LEAF-Q) has been used to assess the risk of LEA in athletes, with the prevalence reported to range between 33 and 46% [19,20]. However, most of the existing literature has focused on endurance athletes, and little is known about team sport athletes. Furthermore, female Australian rules football players may be at an increased risk of low EA due to an increase in energy expenditure after joining a professional program. Thus, screening for the risk of LEA in this group of athletes may have important long-term implications for health and performance and may determine the need for further education to ensure appropriate dietary intake to meet the demands of the sport.

To date, no previous studies have assessed dietary intake, nutritional knowledge or risk of low energy availability in female Australian rules football players. Further, data assessing nutritional adequacy in Australian female athletes, in particular team sport athletes, are limited. As the AFLW league is new and rapidly growing, this information will be beneficial for coaches, fitness trainers, medical staff, nutritionists and dietitians working with these female athletes. Thus, the aims of the current investigation were to: (a) quantify energy, macronutrient and micronutrient intake in female Australian rules football players and compare these to current recommendations, (b) quantify sports nutrition knowledge in female Australian rules football players, and (c) quantify the risk of low EA in female Australian rules football players and compare nutrient intake between players who are at risk of low EA and those not at risk.

## 2. Materials and Methods

### 2.1. Subjects and Study Design

This study followed a cross-sectional design, involving VFLW players (n = 30) aged 18–35 years. Players were recruited in Victoria from one elite-level professional AFL club. Data collection was performed during the 2017 VFLW preseason (March–April). The study was conducted in accordance with the Declaration of Helsinki, and the Deakin University Human Research Ethics Committee approved the study (Project Approval Code: EC00213). All players provided their written informed consent prior to their involvement in the study.

### 2.2. Data Collection

#### 2.2.1. Anthropometry

Height (cm) was measured using a portable stadiometer (Seca, 213, Hamburg, Germany), and body mass (kg) was measured using electronic scales (A&D Australasia, HW-PW200, Adelaide, South Australia) by a qualified research assistant.

#### 2.2.2. Nutritional Intake

The Automated Self-Administered 24 Hour (ASA24) Dietary Assessment Tool [21], based on an Australian-based food and beverage database, was used to assess the previous 24 h (midnight to midnight) nutritional intake on three non-consecutive training days within a seven-day period. The data entered were checked via a follow-up interview with a qualified nutritionist. The mean daily intake of energy, macronutrients and micronutrients over the three 24 h recalls was calculated. The mean daily protein and carbohydrate intake was compared with the values reported in the current American College of Sports Medicine (ACSM) sports nutrition guidelines [6,22] and were expressed in grams (g), relative to BM (g⋅kg⋅BM^−1^). Carbohydrate intake was compared to relevant recommended values (5–7 g⋅kg⋅BM^−1^ and 6–10 g⋅kg⋅BM^−1^) [6], given the physical demands and duration of pre-season training. Daily fat intake, expressed as a percentage of total daily energy intake, and micronutrient intake, expressed as milligrams (mg) or micrograms (µg), was compared with those established by the Australian general population health recommendations [10] as there are currently no guidelines specific to athletes. The current type of supplement use was captured via an online software program (Qualtrics, Provo, UT, USA).

#### 2.2.3. Sports Nutrition Knowledge

Players completed the 88-item Sports Nutrition Knowledge Questionnaire (SNKQ) [23] via an online software program (Qualtrics, Provo, UT, USA). The SNKQ has been assessed for validity (content and construct) and reliability (test–retest), with findings indicating a high construct validity and good test–retest concordance and therefore suitability to be used to determine sports nutrition knowledge. The SNKQ consists of five sub-sections (general nutrition concepts, fluid, recovery, weight control and supplements). One point was awarded for each correct answer, and an ‘unsure’ or incorrect response received zero points. The overall score (out of 88) was expressed as a percentage of correctly answered questions.

#### 2.2.4. Energy Availability

The 25-item LEAF-Q was used to assess the risk of LEA considering three factors: gastrointestinal function, injuries and menstrual function. The LEAF-Q has been validated in female athletes aged 18–39 training ≥5 times/week, with findings producing an acceptable sensitivity (78%) and specificity (90%) to classify current energy availability [16]. Consistent with the original validation study [16], players completed a paper version of the LEAF-Q to ensure validity and reliability were maintained. Scoring was based on the original validation study, with those who scored ≤7 being classified as ‘not at risk’ of LEA, and those who scored ≥8 being classified as ‘at risk’ of LEA [16].

### 2.3. Statistical Analysis

All statistical analyses were conducted using the Statistical Package for Social Sciences (SPSS 24.0, Chicago, IL, USA). Results were reported as mean ± standard deviation (SD) for normally distributed variables and median and interquartile range (IQR) for non-normally distributed variables. Independent samples *t*-tests were used to compare energy and macronutrient intake between players who were deemed to be at risk of low EA (≥8) and those who were shown to be not at risk of low EA (≤7). Statistical significance was set to a level of *p* ≤ 0.05.

## 3. Results

The demographic characteristics of players (n = 30) are provided in Table 1. The completion rate of the ASA-24 dietary recalls, SNKQ and LEAF-Q was 29 (97%), 30 (100%) and 27 (90%), respectively, for 30 participants.

Nutritional intake is presented in Table 2. Mean carbohydrate intake was below recommendations for training days (moderate- to high-intensity activity), while protein and fat intake met current recommendations (Table 2). In comparison with current sports nutrition guidelines, carbohydrate intake was less than the recommended value for moderate exercise of approximately one hour (5–7 g⋅kg^−1^) in 96.3% of players and for moderate to intense exercise (6–10 g⋅kg^−1^) for 1–3 h [6] in 100% of players.

Micronutrient intake was consistent with current general population guidelines, with the exception of iron and calcium, while sodium intake exceeded the current recommendations (Table 3). In comparison to the general population guidelines, 65.5% of players’ calcium intake was below recommendations, while none of the players met the minimum requirement for iron. Despite the average intake recommendations, 38% of players’ magnesium and 20.7% of players’ zinc was below the general population guidelines.

The median score for total sports nutrition knowledge was 48 correct answers out of possible 88 (54.5%) [23]. The lowest median scores were observed in the sub-section on supplements (18% correctly answered) (Table 4).

The median score on the LEAF-Q was 7, with 8 out of 27 players (30%) scoring above 8, indicating a risk of LEA [16]. Energy and macronutrient intake did not differ (*p* > 0.05) between players who were classified as at risk of LEA and players who were classified as not at risk of LEA (Table 5). Table 6 highlights the key LEAF-Q responses. Many players reported injuries in the previous year, the most common being ankle- or foot-related. Nine of the 27 players (33%) reported exercise-induced changes to menstrual function. Gastrointestinal symptoms not related to menstruation were less frequent.

**Figure 1:**
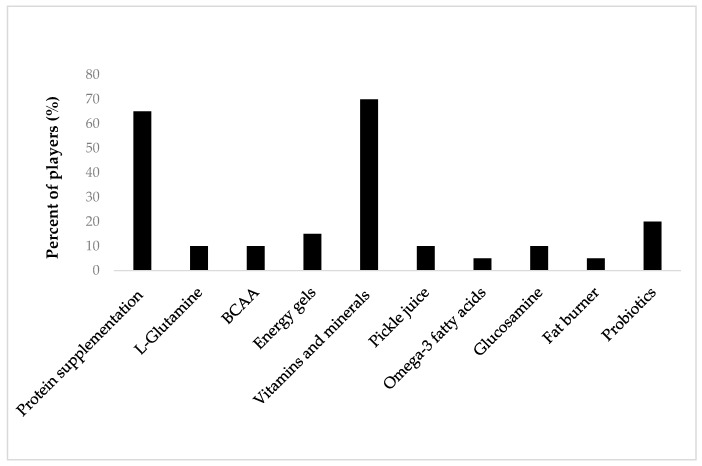
Current supplement use in VFLW players (n = 30). BCAA: branched-chain amino acids

The most common forms of supplementation used by players were protein supplementation (65%) and vitamins and minerals supplementation (70%) (Figure 1). Performance-enhancing supplements such as creatine, B-alanine, caffeine and nitrates were not used by any player (Figure 1).

## 4. Discussion

This is the first study to assess nutrient intake, sports nutrition knowledge and risk of LEA within female Australian rules football players. The key findings from this study were that carbohydrate intake on training days was below recommendations for players performing moderate training of approximately one hour per day (5–7 g⋅kg^−1^⋅d^−1^) and moderate- to high-intensity training of 1–3 h per day (6–10 g⋅kg^−1^⋅d^−1^) [6]. Protein intake was consistent with current recommendations (1.2–2 g⋅kg^-1^⋅d^−1^) [6] and consistent with previous findings [5,7,8]. The assessment of micronutrient intake showed that the average intake of calcium and iron was below the general population recommendations for females (1000 mg⋅d^−1^ and 18 mg⋅d^−1^, respectively), while other micronutrients were in line with recommendations. Sodium intake exceeded the recommended daily intake for the general population (460–920 mg⋅d^−1^). The overall sports nutrition knowledge scores were low, with the lowest level of knowledge related to supplementation (18% correctly answered). Furthermore, 30% of players were deemed to be at risk of LEA, which is similar to previously reported rates in female athletes [19,20].

Mean energy intake in this group of female Australian rules football players was 7826 ± 2411 kJ⋅kg^−1^, which is slightly below that reported in previous studies in Australian female athletes, with mean energy intakes ranging from 8674 kJ to 10,511 kJ⋅kg^−1^ [9,24,25]. The lower energy intake in the current investigation may be due to the nature of Australian rules football. Endurance athletes (such as cyclists and triathletes) have been reported to have higher energy intakes (11,850 kJ per day) than team sport athletes such as softball players (8959 kJ per day) and power sport athletes within individual sports such as track and field (8951 kJ per day) [9]. Furthermore, the method of estimating energy intake may also contribute to the differences in results, with previous studies using food frequency questionnaires (FFQ), which can overestimate energy intake [9], or 24 h recalls, which can underestimate energy intake [24]. In the current study, the repeated online 24 h recalls may have underestimated energy intake, since the ASA-24 is a self-administered tool. The estimation of energy requirements in football players is challenging, as requirements can increase or decrease depending on age, athlete caliber, overall daily activity levels and body composition [26]. Objective measures of energy expenditure such as those obtained by using accelerometers can provide a more accurate representation of energy requirements; however, there are limitations to their use in high-intensity sport as well as when performing resistance training [18,27]. To overcome differences in exercise modes, the use of weighed food diaries has been suggested as the gold standard for assessing dietary intake in athletes [9]. Therefore, further research is required to estimate energy expenditure and intake using the most accurate tools currently available, in order to more comprehensively determine whether energy intake within this population is sufficient.

Carbohydrate intake assessed on training days (involving a minimum of one hour of moderate exercise) in this group of VFLW players was below the minimum recommended 5 g⋅kg^−1^⋅d^−1^ [6]. This finding is consistent with previous findings in both Australian female athletes [9,24] and male AFL players [5,7,8]. Furthermore, the majority of players (96.3%) did not meet the recommendations for athletes performing moderate exercise for approximately 1 h per day (5–7 g⋅kg^−1^), while no players (0%) met the recommendation for moderate- to high-intensity exercise for 1–3 h per day (6–10 g⋅kg^−1^) [6], consistently with previous reports of carbohydrate intake during pre-season training [5,28]. Female athletes from a range of different sports have previously been reported to have a similar daily intake of carbohydrate to that of the current investigation (4.5 g⋅kg^−1^⋅d^−1^), with the lowest intakes in softball players (3.3 g⋅kg^−1^⋅d^−1^) [9]. Furthermore, it was reported that female endurance athletes’ carbohydrate intake was 6.7 g⋅kg^−1^⋅d^−1^, higher than that for other female athletes [9], a result which may have been due to either a higher training demand or a greater knowledge of the importance of carbohydrate for performance in this sport [9]. Low-carbohydrate diets have become popular amongst the general public as well as female athletes, as they are often perceived to be a tool for weight or fat loss [29]. In athletes, a sustained low-carbohydrate intake may be insufficient to support the training demands, possibly impacting on performance and long-term health [29]. The carbohydrate intakes observed in the current investigation, however, are consistent with those previously reported for both male and female team sport athletes [30]. Furthermore, in the current study, there was no significant difference in carbohydrate intake between players who were at risk of LEA and those not at risk, suggesting that the carbohydrate intake in this group may be sufficient to meet the training demands. The current carbohydrate intake recommendations are based on the outcomes of studies that include athletes from varied populations, with a large proportion from endurance sport [6], and therefore it should be considered whether they are applicable to team-based sports, which often involve intermittent and skill-based training [31]. Thus, further research in female team sport athletes’ habitual carbohydrate intake, glycogen depletion rates and optimal carbohydrate periodization interventions [32,33] is required to provide evidence for guidelines specific to a female team sport athlete population.

The mean intake of most micronutrients in the current investigation was above the Australian RDI for the general population. Only one other Australian study has measured micronutrient intake in female athletes, reporting similar findings [9], the key differences being that within the current study, calcium (924.8 mg⋅d^−1^) and iron (12.2 mg⋅d^−1^) intakes were below the RDI, and a higher proportion of athletes did not meet the RDI for calcium (65%) and iron (100%). Adequate intakes of calcium, which plays a vital role in bone health and injury risk [34,35], and iron, essential to carry oxygen and maintaining energy release [36], are important for females, in particular when performing high-intensity contact sport. The main sources of calcium in the diet are dairy foods, including milk, cheese and yoghurt [10], while the main sources of iron include meat products, in particular red meat [10]. In the current investigation, the mean intake of dairy food per day was 1.2 serves, which is below the recommended 2.5 serves for the age group of the participants [10]. Furthermore, 17% of players did not consume any dairy food, and only 53% of players reported drinking milk, while only 50% consumed red meat 2–4 times per week. A low intake of dairy [37] and meat [36] foods is commonly reported in young Australian females and may be a result of the perception that these foods can contribute to weight gain [38,39]. Furthermore, intolerances to dairy food and lactose products have become more common [40], and veganism has become more visible and accepted in the sports industry [41], possibly contributing to the low intakes of these minerals. Nonetheless, there is a need for further education focused on the importance of calcium intake and the appropriate dietary sources for female football players.

Sodium intake in the current investigation was substantially higher than the recommended intake for the Australian general population. In the current study, 20% of players reported consumption of sports drinks, which are typically high in sodium, on a daily basis, which may therefore have contributed to the overall sodium intake [42]. There is limited available evidence on the required sodium intake in an athletic population, as there is a high degree of inter-variability between athletes, often dependent on individual sweat sodium concentrations and sweat rates [43]. Furthermore, the current recommendations are aimed at reducing the risk of chronic disease [10], and a recent study found that the general health recommendations of a low-sodium diet may not be appropriate for athletes, especially in warm conditions [44]. Overall, there is limited understanding of the micronutrient requirements of athletes and whether their training demands can be adequately met by intakes that match the general population guidelines. For female athletes, menstruation may affect micronutrient status, especially that of iron, vitamin B12 and zinc, which is another consideration that warrants further investigation.

The results of the current investigation suggest that female Australian football players do not have a high level of sports nutrition knowledge. This is the first study to investigate sports nutrition knowledge within the female population of Australian rules football players; however, the median score for overall sports nutrition knowledge (54.4%) was similar to that previously reported in male Australian football players [7,14]. The aforementioned similarity in sports nutrition knowledge was found despite the previous studies’ use of a different sports nutrition knowledge questionnaire [45]. In a recent study from our group using the same sports nutrition knowledge questionnaire as that used in the current investigation [46], we found scores to be 51% in both elite and sub elite male Australian football players [8]. These observations collectively suggest that sports nutrition knowledge levels may be similar between male and female Australian rules football players. Improving upon low sports nutrition knowledge could be beneficial to male and female Australian rules football players, as it has recently been suggested that for team sport athletes, an increased level of sports nutrition knowledge might facilitate improvements in performance [47]. Furthermore, in the current investigation, knowledge specific to supplements elicited in the lowest median scores (18%), which is consistent with the result reported for male sub-elite players in our recent investigation [8], suggesting that deficits in specific areas of sports nutrition knowledge may affect both males and female Australian rules football players similarly.

In the current study, 30% of players were deemed to be at risk of LEA. This is the first investigation to explore the risk of low energy availability for female Australian rules football players. However, recent investigations have reported an incidence of LEA of 33.3% in a similar participant population and stage of the training year (competitive female soccer players in either their pre-season or competitive season) [48,49]. In the current study, energy and macronutrient intake did not differ between players who were classified as at risk of LEA and players who were classified as not at risk of LEA. This observation contrasts with the result of a previous investigation [45] which reported significantly lower daily energy intake in females with LEA. The aforementioned study, however, directly measured energy availability, whereas only the risk of LEA was quantified in the current investigation, highlighting the need for the further investigation and direct measurement of EA in female Australian football players. It has been conclusively determined that LEA can induce adverse health effects such as increased injury rates and menstrual dysfunction [50], and indeed, in the current investigation, players who were identified as “at-risk” of LEA reported injuries in the past year. Furthermore, exercise-induced changes to menstrual function were reported in one-third of players in the current investigation. There is at least some positive association between knowledge of appropriate nutritional practices and nutritional intake [51]. Therefore, the implementation of nutritional education programs that incorporate specific information about the potentially deleterious health effects of long-term LEA may assist to improve health outcomes for female Australian rules football players.

Several limitations need to be addressed in the current study. Firstly, the players in this study were recruited from one professional sporting club, and thus the sample size was limited by the number of players in the VFLW team. As a result, additional analysis investigating the correlation between nutritional intake and knowledge could not be included within the current investigation. This data would have provided insight into the potential factors influencing nutritional intake, forming the basis of targeted education programs. However, the aim of this study was to gather detailed dietary information in an emerging athlete population, which future studies can build upon. Furthermore, the findings may be specific to players from this club. However, given that this study was conducted during the early phase of the pre-season, in the inaugural year of the VFLW competition, it is unlikely that the club’s philosophies, training or education programs would have impacted on the players. Although dietary intake data were captured on training days, training logs were not kept, which limited the detail of records in terms of training session duration and intensity. The dietary assessment tool also has limitations, as, although it is validated [21], the online ASA-24 is self-administered and, therefore, subject to memory bias and underreporting. Finally, the LEAF-Q used in this study can only assess the risk of LEA. To objectively and accurately investigate energy availability, gold standard methods to assess energy availability include doubly labelled water, portable metabolic carts and weighed food diaries over numerous days. However, these methods are costly, burdensome on athletes and often impractical in field-based studies. Other methods to assess energy expenditure such as the use of accelerometers and weighed food diaries (used for a limited number of days) may be more realistic to implement for athletes and would provide further information on the status of energy availability.

## 5. Conclusions

The results of the current investigation suggest that the female population of Australian rules football players exhibits inadequate carbohydrate, iron and calcium intake with respect to the current recommendations for athletes. Almost one-third of players may be at risk of LEA, and therefore the prevalence of LEA within this population may be similar to that reported for other female athletes. Protein intake was in line with athlete recommendations, while sodium intake within this athlete population appeared to exceed the recommended intake for general populations. Overall, sports nutrition knowledge was found to be low for female Australian football players, especially regarding supplementation. Female football players may benefit from education programs that provide details of the deleterious effects of prolonged LEA.

## Figures and Tables

**Table 1:** Demographic characteristic of VFLW players (n = 30).

	(n = 30)
(Mean ± SD)	
Age (years)	24.15 (±4.1)
Mass (kg)	64.5 (±8.0)
Height (cm)	168.2 (±7.6)
**Birth Place [N, (%)]**	
Australia	30 (100)
**Relationship Status [N, (%)]**	
Married	3 (10.0)
De facto	0 (0.0)
Committed dating/Engaged	13 (43.3)
Never married/Single	14 (46.7)
**Level of Education [N, (%)]**	
Year 12 or equivalent	13 (43.3)
Trade/apprenticeship	1 (3.3)
Certificate/diploma	4 (13.3)
University degree	9 (30.0)
Higher University degree	3 (10.0)

VFLW: Victorian Football League Women’s; SD: standard deviation; N: number.

**Table 2:** Macronutrient intake of VFLW players (mean ± SD) and current recommendations.

Nutrient Intake	VFLW (n = 29)	Recommendation	Percent Meeting Minimum Recommendation
Energy, kJ Energy kJ⋅kg^−1^⋅d^−1^	7826 ± 2411.6 199.5 ± 37.4	NA	NA
Protein, g Protein, g⋅kg^−1^⋅d^−1^	98 ± 32.1 1.5 ± 0.5	NA 1.2–2.0 ^a^	NA 77.8%
Carbohydrate, g Carbohydrate, g⋅kg^−1^⋅d^−1^	192.4 ± 51.8 3.0 ± 0.8	NA 5–7 ^b^ 6–10 ^c^	NA 3.7% 0%
Sugar, g Sugar, % of E	86.2 ± 33.1 18.6 ± 4.4	NA NA	NA NA
Fibre, g	25.5 ± 8	25g ^d^	43.8%
Total fat, g Total fat, % of E	72.2 ± 33.4 33.2 ± 6.5	NA 20–35 ^d^	NA 96.4%
Saturated fat, g Saturated fat, % of E	25.7 ± 14.6 11.6 ± 3.2	NA <10 ^d^	NA 34.4%
Monounsaturated fat, g	29 ± 14.1	NA	NA
Polyunsaturated fat, g	11.4 ± 4.8	NA	NA

% of E: percentage of total energy intake; NA: Not Applicable. ^a^ source: reference [6], ^b^ source: reference [6], up to 1 h of moderate exercise/day ^c^ source: reference [6], 1–3 h of moderate- to high-intensity exercise/day, ^d^ source: reference [10].

**Table 3:** Micronutrient intake of VFLW players (mean ± SD) and current recommendations.

Nutrient Intake	VFLW (n = 29)	Recommended Dietary Intake (RDI) or Adequate Intake (AI) ^a^	Upper Limit	Percent Meeting Minimum Recommendation
Calcium, mg	924.8 ± 544.7	1000	2500	34.5%
Iron, mg	12.2 ± 3.2	18	45	0%
Magnesium, mg	367.5 ± 137.8	310	350 (as a supplement)	82.8%
Phosphorus, mg	1569.3 ± 549.4	1000	4000	96.6%
Potassium, mg	3109 ± 1173	2800	NA	58.6%
Sodium, mg	2063.3 ± 957	460–920	Not Determined	100%
Zinc, mg	11.7 ± 4	8	40	79.3%
Selenium, μg	98.1 ± 64.7	60	400	79.5%
Vitamin C, mg	106.8 ± 115.3	45	NA	82.8%
Thiamine, mg	1.9 ± 1.9	1.1	NA	69.0%
Riboflavin, mg	2.8 ± 2.2	1.1	NA	96.6%
Niacin, mg	25.5 ± 8.9	14	35	96.7%
Folate, μg	484.6 ± 149.8	400	1000	69.0%
Vitamin B12, μg	13.7 ± 46.8	2.4	NA	93.1%

^a^ source: reference [10].

**Table 4:** Nutrition knowledge scores in VFLW players (median (IQR), percent (%) of correct answers).

Sections (No. Questions)	VFLW (n = 30)
	Median (IQR), %
**General Nutrition Concepts (46)**	28 (7), 60.8%
**Fluid (9)**	6 (7), 66.7%
**Recovery (7)**	4 (3), 57.1%
**Weight Control (15)**	7 (3), 46.7%
**Supplements (11)**	2 (3), 18.2%
**Total Nutrition Knowledge (88)**	48 (12), 54.5%

IQR: Interquartile range.

**Table 5:** Energy and macronutrient intake (mean ± SD) in VFLW players at risk of LEA and in players not at risk of LEA (n = 27).

	Not at Risk of LEA (LEAF-Q ≤ 7) (n = 19)	At Risk of LEA (LEAF-Q ≤ 8) (n = 8)	*p*-Value
**Energy (kJ)**	7734.7 ± 2192.4	7699.3 ± 2552.6	0.971
**Protein (g·kg^−1^·d^−1^)**	1.5 ± 0.4	1.7 ± 0.8	0.296
**Carbohydrate (g·kg^−1^·d^−1^)**	2.9 ± 0.8	3.4 ± 1.1	0.248
**Fat (g)**	73.1 ± 30.1	63.9 ± 31.7	0.490

LEA: Low Energy Availability; LEAF-Q: LEA in females Questionnaire.

**Table 6:** Responses to key components of the LEAF-Q (n = 27).

Leaf Questionnaire Component	Frequency (N)	Percent (%)
1. Injury history		
Number of days lost from participation due to injury in past year:		
0	11	40.7
1–7	6	22.2
8–14	4	14.8
15–21	3	11.1
≥22	3	11.1
Most common injuries reported (respondents could choose more than one)		
Back injury	3	11.1
Knee injury	2	7.4
Head injury/concussion	4	14.8
Groin injury	2	7.4
Shin splints/calf tightness	4	14.8
Ligament/tendon injury of the thumb	4	14.8
Rolled ankle/Achilles soreness/broken foot	6	22.2
Illness	2	7.4
Hamstring strain	1	3.7
Hip injury	1	3.7
Shoulder injury strained ac joint	2	7.4
2. Menstrual function exercise-related menstrual changes:		
Bleed less	3	11.1
Menstruation stops	4	14.8
Increased bleeding	2	7.4
Number of periods in the last year (n = 21) (if still menstruating)		
≥9	19	90.5
5–8	0	0
0–2	2	9.5
3. Gastrointestinal disturbances abdominal bloated/gaseous when not having periods:		
Daily-weekly	2	7.4
Seldom	12	44.4
Rarely or never	13	48.1
Cramps/stomach ache not related to your menstruation:		
Daily-weekly	1	3.6
Seldom	8	28.6
Rarely or never	19	67.9
